# Health, Psychosocial, and Social Issues Emanating From the COVID-19 Pandemic Based on Social Media Comments: Text Mining and Thematic Analysis Approach

**DOI:** 10.2196/22734

**Published:** 2021-04-06

**Authors:** Oladapo Oyebode, Chinenye Ndulue, Ashfaq Adib, Dinesh Mulchandani, Banuchitra Suruliraj, Fidelia Anulika Orji, Christine T Chambers, Sandra Meier, Rita Orji

**Affiliations:** 1 Faculty of Computer Science Dalhousie University Halifax, NS Canada; 2 Department of Computer Science University of Saskatchewan Saskatoon, SK Canada; 3 Department of Psychology and Neuroscience Dalhousie University Halifax, NS Canada; 4 Department of Pediatrics Faculty of Medicine Dalhousie University Halifax, NS Canada; 5 Department of Psychiatry Faculty of Medicine Dalhousie University Halifax, NS Canada

**Keywords:** social media, COVID-19, coronavirus, infodemiology, infoveillance, natural language processing, text mining, thematic analysis, interventions, health issues, psychosocial issues, social issues

## Abstract

**Background:**

The COVID-19 pandemic has caused a global health crisis that affects many aspects of human lives. In the absence of vaccines and antivirals, several behavioral change and policy initiatives such as physical distancing have been implemented to control the spread of COVID-19. Social media data can reveal public perceptions toward how governments and health agencies worldwide are handling the pandemic, and the impact of the disease on people regardless of their geographic locations in line with various factors that hinder or facilitate the efforts to control the spread of the pandemic globally.

**Objective:**

This paper aims to investigate the impact of the COVID-19 pandemic on people worldwide using social media data.

**Methods:**

We applied natural language processing (NLP) and thematic analysis to understand public opinions, experiences, and issues with respect to the COVID-19 pandemic using social media data. First, we collected over 47 million COVID-19–related comments from Twitter, Facebook, YouTube, and three online discussion forums. Second, we performed data preprocessing, which involved applying NLP techniques to clean and prepare the data for automated key phrase extraction. Third, we applied the NLP approach to extract meaningful key phrases from over 1 million randomly selected comments and computed sentiment score for each key phrase and assigned sentiment polarity (ie, positive, negative, or neutral) based on the score using a lexicon-based technique. Fourth, we grouped related negative and positive key phrases into categories or broad themes.

**Results:**

A total of 34 negative themes emerged, out of which 15 were health-related issues, psychosocial issues, and social issues related to the COVID-19 pandemic from the public perspective. Some of the health-related issues were *increased mortality*, *health concerns*, *struggling health systems*, and *fitness issues*; while some of the psychosocial issues were *frustrations due to life disruptions*, *panic shopping*, and *expression of fear*. Social issues were *harassment*, *domestic violence*, and *wrong societal attitude*. In addition, 20 positive themes emerged from our results. Some of the positive themes were *public awareness*, *encouragement*, *gratitude*, *cleaner environment*, *online learning*, *charity*, *spiritual support*, and *innovative research*.

**Conclusions:**

We uncovered various negative and positive themes representing public perceptions toward the COVID-19 pandemic and recommended interventions that can help address the health, psychosocial, and social issues based on the positive themes and other research evidence. These interventions will help governments, health professionals and agencies, institutions, and individuals in their efforts to curb the spread of COVID-19 and minimize its impact, and in reacting to any future pandemics.

## Introduction

### Background

Infectious diseases have occurred in the past and continue to emerge. Infectious diseases are termed “emerging” if they newly appear in a population or have existed but are increasing rapidly in incidence or geographic range [1]. Examples of emerging infectious diseases include acquired immunodeficiency syndrome, Ebola, dengue hemorrhagic fever, Lassa fever, severe acute respiratory syndrome (SARS), H1N1 flu, Zika, etc [2]. Evidence shows that emerging infectious diseases are among the leading causes of death and disability globally [3]. For instance, a 1-year estimate of the 2009 H1N1 flu pandemic shows that 43-89 million people were infected [4], and 201,200 respiratory deaths and 83,300 cardiovascular deaths were linked to the disease [5] worldwide. In addition, 770,000 HIV deaths were recorded in 2018 alone, with approximately 37.9 million people already infected with the virus globally [6]. Ebola is another deadly infectious disease that has an average case-fatality rate of about 50%, with a range of 25%-90% case-fatality rates in past outbreaks [2,7].

In December 2019, COVID-19, caused by the novel coronavirus, emerged and soon became the latest deadly infectious disease [8,9] worldwide, with more than 9.4 million confirmed cases and over 482,800 deaths in 188 countries and regions as of June 25, 2020 [10]. Hence, it was declared a pandemic by the World Health Organization. The COVID-19 pandemic has strained the global health systems and caused socioeconomic challenges due to job losses and lockdowns (and other restrictive measures) imposed by governments and public health agencies to curtail the spread of the virus. Evidence has already shown that emerging infectious diseases impose significant burden on global economies and public health [3,11-13]. To understand public concern, personal experiences, and factors that hinder or facilitate the efforts to control the spread of the COVID-19 pandemic, social media data can produce rich and useful insights that were previously impossible in both scale and extent [14].

Over the years, social media has witnessed a surge in active users to more than 3.8 billion worldwide [15], making it a rich source of data for research in diverse domains. In the health domain, social media data (ie, user comments or posts on Twitter, Facebook, YouTube, Instagram, online forums, blogs, etc) have been used to investigate mental health issues [16,17], maternal health issues [18,19], diseases [20-24], substance use [25,26], and other health-related issues [27,28]. Other domains (eg, politics, commerce, marketing, or banking) have also witnessed widespread use of social media data to uncover new insights related to election results [29-32], election campaigns [33], customer behavior and engagement [34,35], etc. Regarding the COVID-19 crisis, social media data can reveal public perceptions toward how governments and health agencies worldwide are handling the pandemic and the social, economic, psychological, and health impacts of the disease on people regardless of their geographic locations in line with various factors that hinder or facilitate the efforts to control the spread of the COVID-19 pandemic globally.

In this paper, we apply natural language processing (NLP) to understand public opinions, experiences, and issues with respect to the COVID-19 pandemic using data from Twitter, Facebook, YouTube, and three online discussion forums (ie, *Archinect* [36,37], *LiveScience* [38], and *PushSquare* [39]). NLP is a well-established method that has been applied in many JMIR papers and other health informatics papers to understand various health-related issues. For example, Abdalla et al [40] studied the privacy implications of word embeddings trained on clinical data containing personal health information, while Bekhuis et al [41] applied NLP to extract clinical phrases and keywords from a corpus of messages posted to an internet mailing list. Specifically, we aim to answer the following research questions (RQs):

- RQ1: What are the negative issues (health, psychosocial, and social issues) shared by people on social media with respect to the COVID-19 pandemic?
- RQ2: What are the positive opinions or perceptions of people with respect to COVID-19 and how it is being handled?
- RQ3: How can the negative issues be addressed using insights from the positive opinions and other research evidence?

The methodological approach used in answering our RQs are as follows:

- We apply an NLP approach for extracting opinionated key phrases from COVID-19–related social media comments.
- We uncover various negative and positive themes, representing public perceptions toward the COVID-19 pandemic after categorizing the key phrases. Our results revealed 34 negative themes, out of which 15 were *health-related issues*, *psychosocial issues*, and *social issues* related to the pandemic from the public perspective. In addition, 20 positive themes emerged from our results.
- We recommend interventions that can help address the health, psychosocial, and social issues based on the positive themes and other research evidence. These interventions will help governments, health professionals and agencies, institutions, and individuals in their efforts to curb the spread of COVID-19 and minimize its impact, as well as in reacting to any future pandemics.

### Relevant Literature

Social media has been a rich source of data for research in many domains, including health [42]. Research that uses social media in conjunction with NLP within the health domain continues to grow and cover broad application areas such as health surveillance (eg, mental health, substance use, diseases, and pharmacovigilance), health communication, sentiment analysis, and so on [43]. For example, Park and Conway [44] used the lexicon-based approach to track prevalence of keywords indicating public interest in four health issues— Ebola, e-cigarettes, marijuana, and influenza—based on social media data. Afterward, they generated topics that explain changes in discussion volume over time using the latent Dirichlet allocation (LDA) algorithm. Similarly, Jelodar et al [45] applied LDA to extract latent topics in COVID-19–related comments and used the long short-term memory recurrent neural network technique for sentiment classification. Furthermore, Nobles et al [46] used social media data to examine the needs (including seeking health information) of the reportable sexually transmitted diseases community. Their NLP approach involves extracting the top 50 unigrams from the posts based on frequency and then generating topics using the nonnegative matrix factorization technique instead of LDA. Paul et al [47] applied the Ailment Topic Aspect Model to generate latent topics from Twitter data with the aim of detecting mentions of specific ailments including allergies, obesity, and insomnia. They used a list of key phrases to automatically identify possible systems and treatments. McNeill et al [48] investigated how the dissemination of H1N1-related advice in the United Kingdom encourages or discourages vaccine and antiviral uptake using Twitter data. They conducted an automated content analysis using the KH Coder tool (Koichi Higuchi) to explore potential topics based on frequency of occurrence and then performed a more detailed or conversational analysis to understand skepticism over economic beneficiaries of vaccination and the risks and benefits of medication based on public opinion. On the other hand, Oyebode et al [49] performed sentiment analysis on user reviews of mental health apps using the machine learning approach. They compared five classifiers (based on five different machine learning algorithms) and used the best performing classifier to predict the sentiment polarity of reviews. However, none of the aforementioned approaches considers the context in which words appear in unstructured texts, which instinctively plays a substantial role in conveying meaning.

To investigate the significance of contextual text analysis, Dave and Varma [50] compared the noncontextual n-gram chunking approach and the contextual part-of-speech (POS) chunking approach in their experimental research in the field of advertising. Although the n-gram chunking method simply extracts words of varying lengths within a sentence boundary as candidate key phrases, the POS chunking method infers the context of words using POS patterns such as one or more noun tags (NN, NNP, NNS, and NNPS) along with adjective tags (JJ) and optional cardinal tags (CD) and determiners (DT). They focused on key phrases up to a length of 6 for their experiments. Their initial assessment showed that the majority of the key phrases generated using the n-gram chunking method are not meaningful within the advertising context, hence not useful. Furthermore, they observed the impact of key phrases from both methods on the performance of classification systems based on naive Bayes, logistic regression, and bagging machine learning algorithms. Their findings revealed that systems using the POS chunking method outperformed those using the n-gram chunking method for feature extraction. We leveraged Dave and Varma’s [50] contextual method in this study and extended it to capture additional POS patterns, NLP preprocessing techniques, and sentiment scoring using a lexicon-based technique.

Finally, to uncover insights about the type of information shared on Twitter during the peak of the H1N1 (swine flu) pandemic in 2009, Ahmed et al [51] generated 8 broad themes using a coding method involving expert reviewers. Similarly, Bekhuis et al [41] involved two dentists to manually and iteratively classify clinical phrases into categories and subcategories. We also used this method in the key phrase categorization stage of our study to group related key phrases into categories or broad themes.

## Methods

### Overview

**Figure 1:**
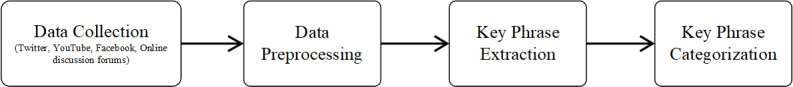
Methodological stages.

The main goal of this paper is to understand and reflect on people’s personal experiences and opinions with respect to the COVID-19 pandemic using social media data. To achieve this, we applied various standard and well-known computational techniques that are highlighted in the following section and summarized in Figure 1.

- We collected COVID-19–related comments or posts from Twitter, Facebook, YouTube, and three online discussion forums using programming languages (Python and C#) and relevant application programming interfaces (APIs).
- We performed preprocessing tasks that involve applying NLP techniques to clean the data and prepare them for the key phrase extraction phase.
- We applied the NLP approach to extract meaningful key phrases, which are words or phrases that convey the topical content of the comments. This approach is in seven stages: *grammar definition*, *sentence breaking and tokenization*, *POS tagging*, *lemmatization*, *syntactic parsing*, *transformation and filtering*, and *sentiment scoring*.
- Based on the sentiment scores associated with the candidate key phrases, we automatically assigned sentiment polarity to the key phrases and then grouped *negative* and *positive* key phrases into categories or broad themes using the thematic analysis method. This helps to answer our RQs.

### Data Collection

We used various automated techniques to collect 47,410,795 COVID-19-related or coronavirus-related comments from six social media platforms: *Twitter*, *YouTube*, *Facebook*, *Archinect*, *LiveScience*, and *PushSquare*. The following describes the techniques and the breakdown of the data collected from each platform:

1. Twitter: We developed a tool using C# programming language to automatically extract tweets containing relevant hashtags in real time through the Twitter Streaming API [52]. To determine trending Twitter hashtags, we searched for “Trending Twitter hashtags on COVID-19” using the Google search engine and retrieved various popular hashtags from several websites including RiteTag [53] and Insider [54]. In addition, we checked a sample of top tweets on Twitter to see other common COVID-19–related hashtags they contained. The selected hashtags were *#CoronaVirus*, *#COVID-19, #Covid_19*, *#COVID19*, *#COVID*, *#QuarantineAndChill*, *#CoronaCrisis*, *#MyPandemicSurvivalPlan*, *#caronavirusoutbreak*, *#CoronavirusOutbreak*, *#Quarantined*, *#pandemic*, *#coronapocalypse*, *#QuarantineLife*, *#StopTheSpread*, *#CoronaVirusUpdates*, *#StayAtHome*, *#selfquarantine*, *#COVIDー19*, *#panicbuying*, *#ncov2019*, *#Coronavid19*, *#SocialDistancing*, *#cronovirus*, *#CoronaVirusUpdate*, and *#CoronavirusPandemic*. A total of 47,249,973 tweets were collected between March 20 and April 3, 2020.
2. YouTube: We developed a Python script to retrieve comments associated with relevant videos through the YouTube Data API [55] using search keywords such as *covid19*, *covid-19*, and *coronavirus*. Due to YouTube’s quota limits, we were only able to extract 111,722 comments across 2939 videos posted between January 1 and April 3, 2020.
3. Facebook: Due to Facebook’s automated search restrictions, we applied a semiautomated approach to extract comments. First, we manually retrieved relevant groups (n=91) and pages (n=68), using the following search keywords: *COVID-19*, *Coronavirus*, and *COVID*. Afterward, we developed a Python script to extract 777 and 8382 comments posted on the groups and pages, respectively, between January 1 and April 3, 2020.
4. Online discussion forums: We developed a Python script to extract 20,747; 793; and 18,401 comments (from coronavirus-related threads) posted on *Archinect*, *LiveScience*, and *PushSquare*, respectively, between January 1 and April 3, 2020.

### Data Preprocessing

Next, we applied the following NLP techniques to clean and prepare data for analysis using Python:

- Remove hashtags, mentions, and URLs
- Expand contractions (eg, *wouldn’t* is replaced with *would not*)
- Unescape HTML characters (eg, “&amp;” is replaced with the “&” equivalent)
- Remove HTML tags (eg, <p>, <span>, and <br />)
- Remove special characters, except those with semantic implications such as periods and exclamation marks (which are useful for identifying sentence boundaries) or commas
- Reduce repeated characters (eg, *toooooool* becomes *tool*)
- Convert slangs to their equivalent English words using online slang dictionaries [56,57], which contain 5434 entries in total
- Remove numeric words

After the preprocessing tasks were completed, non-English and duplicated comments were removed, thereby reducing the total number of comments to 8,021,341.

### Key Phrase Extraction

**Figure 2:**
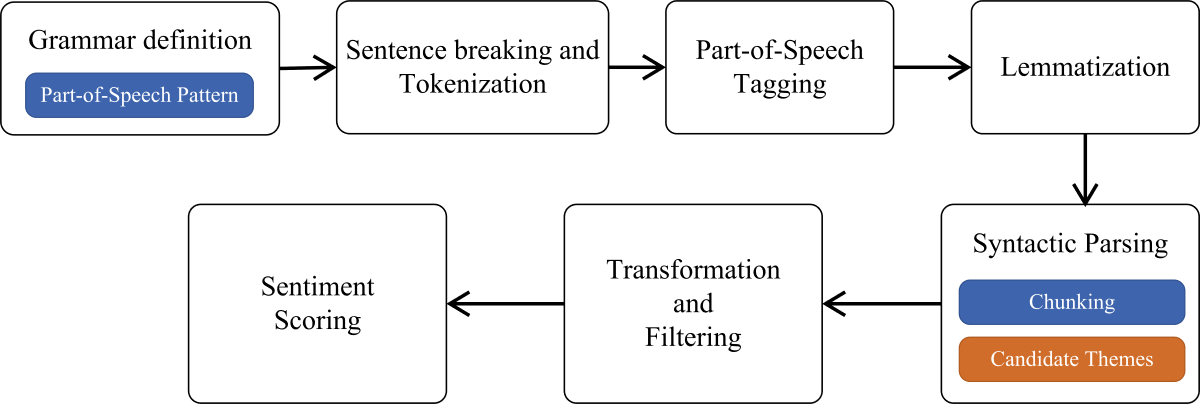
Natural language processing approach.

Next, we randomly selected 1,051,616 comments (representing approximately 13% of the entire data set) and then extracted meaningful key phrases that conveyed the topical content of the comments. We refer to the data set containing the comments as *corpus* and each comment as *document* in the remaining parts of this paper. We focused on key phrases that are opinionated (ie, express or imply positive or negative sentiment [58]) since our goal was to determine public opinions and impact with respect to the COVID-19 pandemic. We extracted candidate key phrases from our corpus using a seven-stage NLP approach, shown in Figure 2. We implemented our approach using the Python programming language.

To derive meaningful key phrases, we defined the following regular grammar: <DT>? <JJ.*>* <NN.*>* <VB.*>? (<IN>? <DT>? <JJ.*>* <NN.*>*)? which specifies a meaningful POS pattern that the syntactic parser uses to deconstruct each sentence in the documents into their constituents [59]. Table 1 shows the various parts of speech (or syntactic categories) captured in the grammar. These syntactic categories are based on well-established POS tagging guidelines for English [60]. In the aforementioned regular grammar, the “?” and “*” characters represent “optional” and “zero or more occurrences,” respectively. Our regular grammar is aimed at generating key phrases that capture both context and sentiment of a conversation using nouns, adjectives, and verbs. Research has shown that nouns are most useful in knowing the context of a conversation (ie, what is being discussed) [61], while verbs and adjectives are important for sentiment detection [62]. Determiners and prepositions are also captured by the grammar since they usually co-occur with noun or adjective phrases (eg, a meal *for* six people or *a* hospital *on the* hilltop).

**Table 1:** Part-of-speech tags, description, and the corresponding matching part-of-speech pattern.

Tag	Description	Matching pattern
DT	Determiner	<DT>
JJ	Adjective	<JJ.*>
JJR	Adjective (comparative)	<JJ.*>
JJS	Adjective (superlative)	<JJ.*>
NN	Noun (singular)	<NN.*>
NNS	Noun (plural)	<NN.*>
NNP	Proper noun (singular)	<NN.*>
NNPS	Proper noun (plural)	<NN.*>
VB	Verb (base form)	<VB.*>
VBD	Verb (past tense)	<VB.*>
VBG	Verb (gerund or present participle)	<VB.*>
VBN	Verb (past participle)	<VB.*>
VBP	Verb (non–third person singular present)	<VB.*>
VBZ	Verb (third person singular present)	<VB.*>
IN	Preposition or subordinating conjunction	<IN>

Next, each document is split into sentences, and then each sentence is split into tokens or words. The sentence breaking task is achieved using an unsupervised algorithm that considers abbreviations, collocations, capitalizations, and punctuations to detect sentence boundaries [63]. The tagging module associates each token with its POS. The POS tags are based on the Penn Treebank tagset [60,64], some of which are shown in Table 1. Each token is reduced to its root form, depending on its POS. This activity is called *lemmatization*. For example, *worse* and *better*, which are both adjectives, will become *bad* and *good*, respectively. Prior to lemmatization, each token is converted to lowercase. Although Witten et al [65] applied stemming for its tokens, we chose lemmatization over stemming since lemmatization returns root words that are always meaningful and exist in the English dictionary. Stemming, on the other hand, may return root words that have no meaning at all since it merely removes prefixes or suffixes based on a rule-based method [66].

Furthermore, the syntactic parsing module deconstructs each sentence into a parse tree and then creates chunks or phrases based on the regular grammar or POS pattern defined in the first step. In other words, the parser’s chunking process involves matching phrases composed of an optional determiner, zero or more of any time of adjective, zero or more of any type of noun, any type of verb (but optional), and an optional component. This component consists of an optional preposition, an optional determiner, zero or more of any type of adjective, and zero or more of any type of noun. The output of this stage is the candidate key phrases.

In the transformation and filtering stage, key phrases that are stop words (ie, words that are commonly used, such as *the*, *a*, *an*, *with*, *in*, and *that*) are removed from candidate key phrases using a predefined list *L_stopwords_* compiled from multiple sources (eg, [67]). We excluded negation words, which are necessary for sentiment detection, such as *not*, from the list of stop words. In addition, a subset of *L_stopwords_* were removed from the start and end of (and from within) the remaining key phrases in the candidate key phrases such that the meaning of the key phrases is preserved. Afterward, duplicates were removed from the candidate key phrases. Although previous research excluded key phrases above length 6 [50], we included key phrases up to length 10 in our analysis to avoid losing important key phrases that would have enriched insights from this paper. Hence, key phrases containing more than 10 words were removed from the candidate key phrases. Since our focus is on opinionated key phrases (ie, positive and negative key phrases), we applied a filtering technique that involves computing sentiment score for each key phrase and discarding nonopinionated key phrases.

Finally, to identify negative and positive key phrases in the candidate key phrases, the scoring module computes a sentiment score, *S_score_*, ranging from –1 to 1 for each key phrase using the Valence Aware Dictionary for Sentiment Reasoning lexicon-based algorithm [68]. Afterward, each key phrase is assigned a polarity (negative or positive) based on the *S_score_* using the criteria recommended by Hutto and Gilbert [68]. Specifically, a key phrase is negative if *S_score_*<–0.05, while a key phrase is positive if *S_score_*>0.05. A neutral key phrase (with *S_score_* between –0.05 and 0.05) was removed from the candidate key phrases since it is not opinionated.

### Key Phrase Categorization

To answer our RQs, we categorized the final candidate key phrases into categories or broad themes using a thematic analysis approach used by Bekhuis et al [41] to classify clinical phrases into categories. In this approach, expert reviewers manually examine the key phrases and then assign them to appropriate categories. We recruited four reviewers to perform our key phrase categorization task. Specifically, we assigned the negative key phrases to a group of two reviewers (G1) and the positive key phrases to a second group of two reviewers (G2). Each reviewer independently examined the key phrases iteratively and continued to categorize related key phrases until a saturation level was reached (ie, no new categories were emerging from the key phrases). Reviewers used coding sheets in which they indicated the category each key phrase belonged to after examining it. Category names were decided by each reviewer such that a new category was created if none of the existing categories matched the key phrase being reviewed. Since key phrases are more specific than comments, the reviewers assigned each key phrase to only one category. In other words, reviewers assign a key phrase to the most appropriate category or to a new category if none of the existing categories was suitable. After categorizing the key phrases, the reviewers in each team validated each other’s work and agreed or disagreed with the category assigned to each key phrase, and offered suggestions to address every disagreement. The reviewers came together after completing their validations to apply the suggestions and ensure all category names were distinct while harmonizing names that are similar. We measured interrater reliability using the *percentage agreement* metric [69]. The percentage agreement score for G1 was 98.0%, while the score for G2 was 99.3%. We refer to the categories as *themes* and the various key phrases under each category as *subthemes* in the remaining part of this paper.

## Results

### Key Phrase Extraction

In this section, we discuss the results of our experiments and key phrase categorization. From the large corpus used for the experiment, 427,875 negative and 520,685 positive key phrases were automatically generated. However, the majority of these key phrases were similar; hence, the reviewers reached a saturation point (during key phrase categorization) where no new categories were emerging. In total, 18,694 negative and 19,841 positive key phrases were categorized.

#### Negative Key Phrases

Multimedia Appendix 1 shows the top 130 negative key phrases and their dominance in terms of frequency of occurrence. Our results revealed that *death* (n=10,187) was the dominant negative key phrase, followed by *die* (n=7240), *fight* (n=5891), *bad* (n=3808), *kill* (n=3668), *lose* (n=3631), *pay* (n=3486), *leave* (n=3234), *crisis* (n=2783), *hard* (n=2720), *worry* (n=2476), *sick* (n=2314), *sad* (n=2129), and so on. More negative key phrases can be found in Multimedia Appendix 2, such as *national health emergency*, *scary time*, *life suck*, *everyone struggle*, *dangerous lie*, *child die*, *trouble breathe*, *no medicine*, *sick people*, *pay bill*, *horrible virus*, *fear coronavirus*, *extra cautious*, *steal mask*, *family die*, *people in crisis*, *bad leadership*, *in house bore*, *feel horrible*, *total incompetence*, *call virus hoax*, *conspiracy theory ridiculous*, *take no precaution*, *serious lockdown*, *increase in suicide rate*, *people starve*, *lack of preparedness*, *fight menace*, and *restriction on travel*.

#### Positive Key Phrases

Multimedia Appendix 3 illustrates the top 130 positive key phrases and their dominance in terms of frequency of occurrence (larger size of the gray oval represents more dominance in the figure in Multimedia Appendix 3). Our results revealed that *help* (n=18,498) was the dominant key phrase, followed by *hope* (n=7708), *protect* (n=7130), *love* (n=6895), *support* (n=6198), *good* (n=5740), *share* (n=5187), *care* (n=4917), *stay safe* (n=4917), and so on. Multimedia Appendix 4 shows more positive key phrases, such as *keep everyone safe*, *clean environment*, *trust scientific data*, *create cure*, *economic relief*, *encourage business*, *remain strong*, *good mask*, *social distancing best way*, *generous*, *respect human right*, *help prevent further spread*, *pray for health*, *social solidarity*, *support relief effort*, *protect health worker*, *good immune system*, *practice good hand hygiene*, *speak truth*, *expand testing*, *protect vulnerable people*, *free treatment*, and *ease anxiety*.

### Key Phrase Categorization

Overall, 34 negative and 20 positive themes emerged after the key phrase categorization phase discussed in the Methods section. Out of the 34 negative themes, 15 were health-related, psychosocial, and social issues (which were the main focus of this paper and are shown in Tables 2-4). Table 5 shows the 15 negative themes and the corresponding number of key phrases under each theme, while Table 6 shows the negative themes and the total number of comments for each theme. Frustration due to life disruptions emerged as the top negative theme with the highest number of comments, followed by increased mortality, comparison with other diseases or incidents, nature of the disease, and harassment. On the other hand, Table 7 shows the 20 positive themes, description, and sample comments. Table 8 shows the corresponding number of key phrases under each positive theme, while Table 9 shows the total number of comments for each theme. Public awareness emerged as the top positive theme based on the number of comments, followed by spiritual support, encouragement, and charity. By identifying negative and positive themes from COVID-19–related comments, we have answered RQ1 and RQ2, respectively.

**Table 2:** Health-related issues: negative themes, descriptions, and corresponding sample comments.

Theme	Description	Sample comments
Increased mortality	Increasing number of deaths due to COVID-19	“Grieving for the world and my country, every night the death count rises up. I cry for everybody that has died, for those people fighting it, & family who have lost someone. I do not know each person by name BUT I want you to know you are not alone in this pain.”^a^ (C58) “...The number of deaths from the Corona-Virus in London are doubling every two days. London could end up with a worse than Italy.” (C90)
Health concerns	Health concerns expressed by people, such as mental health issues (eg, anxiety, depression, stress, or obsessive-compulsive disorder), excessive drinking, migraines, fatigue, and others	“It is been either ten or fourteen days since the nursing home I work at went on lockdown due to Covid_19 and the stress/anxiety is really starting to get to me. I am struggling to sleep at night.” (C3327) “On my fifth day of sickness, the symptoms disappeared, leaving only an odd metallic taste in my mouth, nasal mucosal ulcers and intense fatigue. This is what a former chair of the UK RCGP went through after catching COVID19.” (C945)
Struggling health systems	Inability of health systems to cope with pandemic and give people adequate health care	“What clearly shows is the correlation between countries with clean hospitals and countries with bad hospitals and corona deaths. People die with corona not of corona. In particular New York and California displays its poor health system.” (C81) “Pakistani doctors openly saying their numbers are being underreported. They claim 100s of patients in Lahore alone, and say that because of poor facilities, hospitals themselves might be spreading the virus.” (C44)
Fitness issues	Inability to perform usual physical activity or attend fitness sessions and dislike for indoor workout	“Woke up at 8:20 am and still in bed from the past 2 hours. No mood of workout” (C271) “I just need it to be known that I hate quarantine workouts and I miss the damn gym. Also I never thought I would ever say this in life!” (C209)
Rising number of cases	Concerns over rising COVID-19 cases	“The United States is currently on the path of the most widespread viral attack in the world.” (C908) “This has done a heinous crime on humanity by spreading to more and more areas of the country. The head of the Jamat must be booked for murder crime, as many have died due to Corona infection after attending their function.” (C24)
Nature of disease	Explaining the nature of COVID-19, including its symptoms (eg, cough, loss of taste, and fever), its “no symptom” (or asymptotic) behavior, how it spreads, and vulnerable populations	“Recent reports suggest that Covid-19 does not only affect the respiratory system, but also affects the Central Nervous System. Loss of smell and loss of taste happen to be some of the early symptoms of covid-19.” (C660) “...they are all grabbing and touching the desk. This virus is much more contagious than the flu and there is 0 immunity exposure illness. Keep doing it like this and it is only a matter of time before everyone who spoke will have the virus...” (C900) “If 3 elders died within 72 hours and had no symptoms, should we not be testing everyone?” (C2447)
Comparison with other diseases or incidents	Comparing COVID-19 with other diseases or incidents such as flu, pneumonia, natural disasters, and war	“A war fought with no guns or bombs, where people flee from what they do not see or is it World War 3?” (C515) “I believe Covid19 is this mutated H5N1 avian flu virus. It is airborne, which might explain the rapid spread around the world.” (C671)

^a^All comments are included verbatim, including spelling and grammatical mistakes.

**Table 3:** Psychosocial issues: negative themes, descriptions, and corresponding sample comments.

Theme	Description	Sample comments
Expression of fear	Expression and spread of fear among people, including fear of infection, sickness, and death	“...Indigenous tribes are closing off their reserves to visitors as they fear the disease that is fast spreading across South America could wipe them out...”^a^ (C3884) “...This virus has caused a lot of fear in the lives of many, it has also brought about different mindset in the heart of men. Truly the world is coming to an end.” (C7227)
Retrospection	People recalling past life prior to pandemic and wished they got it back	“I miss football. I miss my family. I miss my friends. But more than all of that, I miss human touch!” (C300) “If you consider yourself and how is social distancing going for you? I miss walking around and reading/writing at local coffee shops.” (C3001)
Frustration due to life disruptions	Expression of frustrations over disruption to everyday life, such as consistent homework (for schoolers), more household chores (for moms), difficulty accessing family members or loved ones, sporting event suspension, postponement of planned trips and tours, higher food prices, and restaurants closure	“Everyday, I wake up from a very normal dream, and realize I have to do another day in this insane world. All I want to do is go see my mom and give her a hug, but I cannot! I feel so alone. I cry every day. I cannot do this much longer.” (C2905) “Day 15 of I only leave my house for food and exercise. Living in such extremes is confusing and disorienting for my body. Every time I step outside, I become hungry and start sweating, preemptively.” (C4484) “Not getting a hair cut in February was a terrible idea. I am two hair shades away from looking like an overweight member of BTS” (C89)
Work from home complaints	Complaints about working from home during pandemic, such as distractions/disturbance, psychological stress, pain, and sleep issues	“Is anyone else experiencing leg and knee pain from working from home too much or is it just me? If so, how have you all dealt with it?...” (C824) “Working from home is an epic fail! I am losing it between my child, pets, monkey calls, and renovations. Wake me when this is over.” (C853)
Panic shopping	People stockpiling groceries and other essential goods due to pandemic	“This panic buying is ridiculous! Heart rending! Guess it takes a situation like to show how selfish callously indifferent we really are towards other humans / animals. Have a heart people!” (C779) “And yet there is still no logical reason for clearing the shelves of toilet roll, depriving those who are old infirm or in poverty from accessing such necessities because the self-serving privileged have greedily taken it all away.” (C4)

^a^All comments are included verbatim, including spelling and grammatical mistakes.

**Table 4:** Social issues: negative themes, descriptions, and corresponding sample comments.

Theme	Description	Sample comments
Wrong societal attitude	Blaming people’s wrong attitude as causing or fanning the spread (eg, disobeying instructions from governments and health care professionals and eating bats and wild animals)	“Cannot believe how irresponsible people are being in regard public health. We all have a duty of care to each other, please abide by the social distance rules”^a^ (C4924) “...We are only going to die from our own arrogance if people keep going outside gathering, believing they will never get it or infect others...” (C2557)
Domestic violence	Rise in domestic violence cases in homes	“Of all women murdered with a gun in the US, half are killed by their intimate partners. COVID19 pandemic is causing a rise in domestic violence. Close the gun stores.” (C56) “...a police station in China received 162 reports of domestic violence in February compared to 47 for the same month in 2019. Advocates attribute this rise in cases to the lockdown.” (C100)
Harassment	Harassing and blaming people from certain countries, race, or religion as responsible for the COVID-19	“...stop being so racist against Northeastern state of India. We are not Corona. Get your damn information right. The most affected places with is not Northeast. Instead of being a racist and criticising others, why do not you be more careful?” (C1413) “An Asian American couple in Minnesota found a racist note taped to their front door blaming them for the coronavirus” (C921)

^a^All comments are included verbatim, including spelling and grammatical mistakes.

**Table 5:** Negative themes and the corresponding number of subthemes.

Themes	Subthemes, n
Frustration due to life disruptions	7106
Increased mortality	4938
Comparison with other diseases/incidents	2673
Harassment	870
Panic shopping	799
Health concerns	798
Nature of disease	481
Expression of fear	338
Rising number of cases	200
Work from home complaints	109
Wrong societal attitude	105
Fitness issues	104
Domestic violence	101
Struggling health systems	48
Retrospection	24

**Table 6:** Negative themes and the corresponding number of comments.

Themes	Comments, n
Frustration due to life disruptions	17,535
Increased mortality	9437
Comparison with other diseases/incidents	6040
Nature of disease	1909
Harassment	1335
Health concerns	1191
Panic shopping	1047
Expression of fear	578
Rising number of cases	264
Fitness issues	151
Domestic violence	123
Work from home complaints	113
Wrong societal attitude	111
Struggling health systems	53
Retrospection	25

**Table 7:** Positive themes, descriptions, and corresponding sample comments.

Theme	Description	Sample comments
Gratitude	Appreciating health workers, delivery workers, farmers, pilots, security agents, other frontline workers, and the government for their active roles during the pandemic	“Let us show our appreciation for hard work the health care professionals are doing to save lives at these thought times.”^a^ (C37) “Thank you to all on the Frontline and the Key Workers, keep up the amazing work, you are doing an amazing job, keep our country going. Keep Smiling in challenging times” (C156)
Public awareness	Raising awareness of the public about general safety and control measures to limit the spread of the disease (eg, good hygiene, social distancing, staying at home, face masks, and healthy eating), addressing misinformation, providing travel guidelines, etc	“Practicing good hygiene, like washing your hands often, with soap and water, for at least 20 seconds, is the best way to prevent the spread of Coronavirus...” (C707) “We can help ourselves by keep practicing proper hygiene, assume anyone around us could be positive and we must keep personal distancing, wear protective gear, mask, gloves, etc. Boost our immune system by eating healthy food. Search what kind of vegetable and fruits is best to increase our body's ability to fight the virus. Sanitize groceries before bringing them inside the house...” (C16) “If extremely necessary, stay healthy while travelling by maintaining personal hygiene, cough etiquette and keeping a distance of at least one metre from others. Here are some travel tips from World Health Organization” (C537)
Cleaner environment	Evidence of cleaner environment, including less pollution and good air quality, due to pandemic-related lockdowns	“Coronavirus, making Earth healthy again” (C588) “Coronavirus pandemic leading to huge drop in air pollution” (C664) “Remember the time when we used to share the post that said, ‘We have only 6 months to take action and save the environment, mend your ways or there is no way to save the earth’. Guess what? A virus saved the environment better than the most evolved beings did.” (C992)
Evidence of recovery from disease	Evidence of people recovering from COVID-19 with or without treatment	“A 95-year-old, become the oldest woman in to recover from the novel coronavirus without the need for antiviral treatment after her body showed a great reaction to the disease, doctors say.” (C214) “He was referring to the number of patients treated in the Baptist Healthcare system here. He said the numbers show 90% of their Coronavirus positive patients recover at home.” (C321)
Homemade protective equipment	People’s ingenuity in creating essential protective equipment (eg, face masks and face shields) themselves in their homes or community	“When your missus tells you she put Vodka in her fairy. How was I supposed to know she is trying to make homemade hand sanitizer with the washing up liquid?” (C9335) “82 and counting. Eager volunteers all over the city are stitching face masks to ensure that our community remains protected despite the imminent shortage of PPEs in this pandemic.” (C129)
Online learning	Public engagement in online learning such as schools teaching students/pupils online and self-development by enrolling in online courses covering different domains	“We will all be spending more time at home in the next few months due to coronavirus. I have already registered myself with number of short courses with online learning for the subjects that interest me. This could be a good time to learn something new or sharpen up your skills.” (C1648) “Despite all the news, today is the first day back to school for Florida's students virtually. Distance learning might be the new normal for a while...” (C710)
Connection with family and friends	Spending time with family, friends, and loved ones due to pandemic and lockdown	“Yesterday my mom had a video call with her group of friends instead of going out for their regular meetup. I am proud of my mom. Who said boomers are outdated?” (C7151) “This is not the time to be selfish. This is the time to be more present. I did a roll call this morning, calling all my friends video call, family and those I care about just to check on them...” (C2560)
Entertainment	People accessing entertaining content online or offline, such as watching movies on streaming websites and playing games	“Stay at Home and Stay Safe. Please share me good Amazon Prime or Netflix movies and television shows.” (C93) “Downloading the play station 1 and nintendo 64 emulators on my pc. Can get any game.” (C399) “9th grade Colin has returned and will be playing grand theft auto for 8 hours straight” (C3192)
Charity	Provision of relief packages (including donations, gifts, and fundraising) for individuals, businesses, and hospitals to ease financial burden caused by the pandemic	“Kicking off NeighborsHelpingNeighbors here in RI with the RhodeIsland Hospitality Relief Fund - a fund aiming to help industry members directly affected by COVID19.” (C3623) “The 2 trillion emergency relief package now before the House provides the following: 1,200 checks for those earning less than 75k, plus 500 per child, unemployment benefits for 39 weeks up from 26, unemployment benefits rise by 600/week for 4 months” (C10913) “Zlatan Ibrahimovic has created a fundraiser to gather 1m to help hospitals in Italy working to tackle the coronavirus. The striker has donated €100,000 to get the fund started.” (C24)
Advocacy of increased testing	Advocacy of increased testing as a means of curbing the spread by detecting infected people and isolating them quickly	“Studies in Iceland show that half of carriers show no symptoms. Having widespread test allows them to isolate those with the virus, and thus, the virus itself. Test, test, test.” (C1333) “US surpassing all countries in number of patients. Is it simply because they are now testing at a faster rate even on slightest suspicion? Yes, one of the ways to stop this pandemic is Testing, Testing and Testing as recommended by WHO” (C6363)
Grassroots support	Extending support to people at the local or community level during the pandemic	“The amazing QueerCare are offering support to local mutual aid groups. They have trained volunteers and are already in contact with people who will need support over the weeks and months to come...” (C2839) “My self, and some other good citizens residing in Lagos bought 150 pieces of pocket hand Sanitizers to distribute to people who cannot afford it or have knowledge of what is all about and in same process to tell them how to stay safe. That is our own giveaway.” (C773)
Access to necessary tools	Access to tracking or communication tools/features for information dissemination or remote communication during pandemic and lockdown	“This is the most stunning visualization of how spread around the world. A mesmerizing and terrifying display of globalization and virus spread.” (C6625) “I would like to personally thank for the private chat function during Zoom video meetings.” (C2244) “This tool is useful in identifying those who are medically more at risk of suffering complications from COVID19...” (C9233)
Spiritual support	Offering prayer of recovery for those with pandemic-related health conditions and those at risk, as well as a show of hope in challenging times	“I pray for everyone diagnose of COVID19 swift and complete recovery in Jesus name. Dear Lord, heal the world. Take away and give everyone good health.” (C9230) “Let’s pray for all African communities who live in hostels, huts, rural areas with no connectivity. No information. Innocents but will also be affected by the CoronaVirus” (C2104) “...those who have relationship with God are less likely to become depressed than those who do not. It is because their confidence and hope is in Him so, let us trust God amid the pandemic.” (C1349)
Solidarity for frontline workers	Public call for support and protection of frontline workers such as health workers and delivery workers	“What is New York City doing to protect the workers at Amazon fulfillment center? The virus is spreading quickly among the employees...” (C3332) “While the news of is getting more urgent by the hour, it is great to know that during a turbulent time some corporations showed their support by increasing the minute wage for frontline workers, hiring 100s of people to assist with increased demand, helping unite.” (C4439)
Development of curative solutions or treatments	Ongoing efforts by health researchers to develop vaccines or drugs to cure or treat COVID-19	“There is a bunch of solutions being researched from I guess infusions to drugs to slow virus reproduction, to vaccines. None will be ready until a year or two. Clinical studies take time and that is how we do medical science safely.” (C5759) “Coronavirus vaccine clinical trial starts Monday, U.S. official says…But officials still say it will take a year to 18 months to fully validate any potential vaccine.” (C2034)
Physical activity	Efforts made by people to stay active and fit, as well as physical activity suggestions, during lockdown or isolation	“I know it is a bad situation but damn I am improving my fitness during this lockdown” (C6335) “Just finished our fitness session via skype with friends. I live in Barcelona, lost track of how many days since I went outside, we have to keep the body moving though” (C9430) “Get active every day with the kids or by yourself! GoNoodle is here to help!...” (C9364)
Encouragement	Encouraging people to stay calm as they cope with the pandemic situation, encouraging people to view the pandemic from a positive standpoint and stay productive amid the challenges, encouraging people to help others in need and not panic buy, and encouraging people to obey lockdown rules and guidelines released by governments and health professionals	“Let us all stay calm. Give the authorities time to attend to and address public concerns...” (C3) “Yes, we can. If we stay calm and respect the rules, together we will defeat the enemy.” (C38) “Stay calm and help each other. Be careful. Do not panic buy, and never give up!” (C164) “We have done it before and can do it now. See the positive possibilities. Redirect the substantial energy of our frustration and turn it into positive, effective, unstoppable determination for our safe and healthy future” (C2273)
Support for remote working	People’s support for the work from home measure, including adapting/coping with the challenges it brings	“Working from home with the children of school can be challenging. We have taken a look at some of the ways you can help structure your day and stay on top of working from home with our schools closed.” (C148) “Working from home amid Coronavirus pandemic was amazing at least today. Came up with some amazing designs...We are coming up with our first property in June. Already sold out” (C53)
Innovative research	Global research efforts to create innovative products to address the pandemic, including developing interventions (eg, digital or technological interventions) that help people socially, physically, emotionally, or psychologically and to improve their overall health and wellness	“Doctors and scientists...have designed an application to help the public monitor their symptoms and the spread of the virus in real-time with the contributing to their own vital research” (C96) “...Well, it is a huge scientific discovery! Scientists want to use artificial intelligence technology for a quicker and cheaper COVID-19 screening...” (C197) “The COVID19 Global Hackathon is an opportunity for developers to build software solutions that drive social impact, with the aim of tackling some of the challenges related to the current coronavirus pandemic...” (C619)
Traditional remedy	Some suggestions regarding the natural or traditional means of protecting the body from contracting the disease	“Gargling vitamin c, vinegar, warm water, and a little bit of baking soda every 20 minutes. After 5 days, she tested negative. If you or anyone you know starts getting symptoms, this can help! Catch it early before it gets to your lungs!” (C803) “...I am sure I have Covid_19. I believe the natural healing helped my daughter but suppressed my symptoms...” (C2777)

^a^All comments are included verbatim, including spelling and grammatical mistakes.

**Table 8:** Positive themes and the corresponding number of subthemes.

Themes	Subthemes, n
Public awareness	8129
Spiritual support	4139
Encouragement	4033
Entertainment	688
Gratitude	670
Charity	657
Development of curative solutions or treatments	587
Advocacy of increased testing	296
Cleaner environment	214
Evidence of recovery from disease	141
Physical activity	71
Connection with family and friends	61
Online learning	46
Access to technology tools	36
Innovative research	19
Grassroots support	17
Homemade protective equipment	14
Support for remote working	10
Solidarity for frontline workers	7
Traditional remedy	6

**Table 9:** Positive themes and the corresponding number of comments.

Themes	Comments, n
Public awareness	22,749
Spiritual support	12,130
Encouragement	5244
Charity	942
Entertainment	798
Gratitude	758
Development of curative solutions or treatments	653
Advocacy of increased testing	341
Physical activity	285
Cleaner environment	278
Evidence of recovery from disease	156
Connection with family and friends	70
Online learning	52
Access to technology tools	52
Innovative research	21
Grassroots support	17
Homemade protective equipment	14
Support for remote working	10
Traditional remedy	9
Solidarity for frontline workers	7

## Discussion

### Principal Results

In this paper, we analyzed social media comments to uncover insights regarding people’s opinions and perceptions toward the COVID-19 pandemic using an NLP approach. Our empirical findings revealed negative and positive themes (see Tables 2-4 and Table 7) representing negative and positive impacts of the COVID-19 pandemic and coping mechanisms on the world population. To answer RQ3, we first discussed each of the negative issues (supported by research evidence) in this section and then suggest interventions to address the issues in a later section.

#### Negative Issues Surrounding the COVID-19 Pandemic

Tables 2-4 show the negative themes grouped under health-related issues, psychosocial issues, and social issues from our results. The health-related issues included *health concerns*, *increased mortality*, *struggling health systems*, *fitness issues*, *nature of disease*, *rising number of cases*, and *comparison with other diseases or incidents*. The psychosocial issues were *expression of fear*, *panic shopping*, *retrospection*, *work-from-home issues*, and *frustration due to life disruptions*. The social issues were *wrong societal attitude*, *domestic violence*, and *harassment*.

##### Health-Related Issues

Evidence shows a rapid increase in the number of COVID-19 cases and a high case-fatality rate of 7.2% [70]. In addition, a substantial number of patients who are infected had severe pneumonia or were critically ill [70]. Another study revealed the mental health issues experienced by people and health professionals directly impacted by the COVID-19 pandemic [71], and the global health care systems’ inability to deal with the outbreak [72]. The themes under this category are discussed in the following subsections. They align with existing research and uncovered additional insights with respect to the health-related issues caused by COVID-19 and witnessed by people worldwide.

###### Health Concerns

Based on our findings, people experienced various mental health issues (eg, anxiety, depression, stress, or obsessive compulsive disorder) during the pandemic. This is possibly due to the length of time spent staying at home (which may be traumatic for some people while causing loneliness for others), worrying about being infected with the disease and difficult living conditions, as well as guilt on the part of health care workers who feel responsible for being unable to save their patients from death. Research confirms that worry is associated with anxiety and depression [73]. Cases of mental health disorders linked to COVID-19 have also been reported [74]. Furthermore, people expressed other concerns like excessive drinking, migraines, chest pains, mild to severe fatigue, nasal mucosal ulcers, sleep disorders, and others. The following are sample comments:

Cannot sleep. Mind is racing. *Feeling* anxious.

C6648

I am *so stressed* and *my anxiety has hit the roof*. I am anxious about money and how we will cope?

C238

This coronavirus outbreak is *more stressful for the family*. Doing my best to keep sanitized and safe. But, fear of the invisible killer lingers on, *taking a mental toll on my mother, wife, son*, who are petrified every time I walk out of the main door.

C116

The *chest pains* today is beyond. It kinda have crawled up a bit and I feel like I put my hand on my heart from time to time. *Very tired* today. But weirdly still no fever. But I am *cold* and I *feel sick*.

C12293

We have only been in for 3 weeks we are already *feeling anxiety, depression severe*, so we decided to think of some ways we can keep ourselves each other in good spirits.

C8263

###### Increased Mortality

People attested to an increase in death rates in many countries across different continents including North America, Europe, Asia, the Middle East, and Australia, as shown in the following sample comments. Many countries, especially those in Africa, started reporting deaths from COVID-19 (see C1264). Our findings also revealed people of varying demographics died from the disease, including teenagers, adults, and older adults, as well as those with or without underlying health conditions (see C8837 and C940).

This is why America *leads the world in the death toll already*, and the pace still is not showing any signs of slackening.

C3399

UK coronavirus death rate DOUBLES as 381 die in 24 hours and boy, 13, with no health problems becomes youngest victim

C8837

Turkish health minister shares latest coronavirus data: *16 more people have died*, bringing death toll to 108

C9000

Kenya has recorded the first death. According to Health Cabinet Secretary,...the 66 years old man died on 26th in the afternoon at the AghaKhan hospital intensive care unit.

C1264

100 more UK deaths in last 24h alone. These are *not all elderly co-morbid people*. Among these are the *young*, and the *fit*...

C940

###### Struggling Health Systems

Health systems worldwide are struggling to cope with the surge in the number of patients with COVID-19 and in most cases are unable to admit patients due to limited resources [75]. Research has shown that health care burden due to COVID-19 is associated with the increase in mortality rate [76]. As revealed in the following sample comments, our findings corroborated evidence of overstretched global health systems during this pandemic.

Hospitals turning away coronavirus patients in California. EMTALA being used to set up tents outside of hospital ER, then dumping patients without treatment or testing. I *obtained recordings of standard hospital practice of rejecting Covid19 patients*.

C4949

These are not just numbers. These are people and families. These lives can be saved if the chunk of $2.2 Trillion are not used for bailing out corporations and used to fix the *broken US Health System*.

C3000

###### Fitness Issues

Evidence argues that the prevalence of physical inactivity worldwide due to nationwide quarantines or lockdowns [77]. This was confirmed in our findings, which showed that people have trouble staying fit due to an inability to control eating habits or urges while at home and have a personal dislike for indoor-only workouts, as shown in the following comments. Physical inactivity has been linked to coronary heart disease, diabetes, stroke, and mental health issues [78-80], which, in turn, are risk factors for mortality in COVID-19 adult inpatients [81].

...severely missing my gym, missing routine, and cannot control my eating while at home. Things are getting bad.

C9002

During this shelter in place, I was gonna eat healthy and kill some workouts. *But instead I've been Guy Fieri'ing around the kitchen sampling all my quarantine food every 2 hours*. At this point, which will get me first - coronavirus or a coronary?

C7182

###### Nature of Disease

People expressed their opinion about the nature of COVID-19 based on their experiences and information available to them. As shown in the following sample comments, people with underlying health conditions (eg, diabetes or heart disease) are at higher risk of developing severe complications from the disease. In addition, the asymptotic attribute of COVID-19 is also discussed, and the possibility of the virus infecting some critical immune cells that may lead to the failure of sensitive organs like the lungs. People also perceived the disease as racial- or nationality–independent but seems to pose more risk to men than women. The disease is also seen as highly contagious and shows symptoms such as cough, fever, fatigue, loss of smell, muscle aches, and respiratory-related symptoms (eg, shortness of breath). These findings align with clinical evidence regarding COVID-19 [82-87].

Some WTC Health Program members with certain health conditions...may be at *a higher risk of serious illness* from COVID19

C3620

...Majority *do not show symptoms* while spreading Covid_19

C10033

...If the coronavirus *infects some of the immune cells* then there is a cellular catastrophe and *organs like lungs are gone within hours*.

C9200

Coronavirus is a disease that *pays no attention to borders, race or nationality*. However, it appears COVID-19 does *pose a noticeably bigger threat to men than it does to women*.

C1902

This is absolutely true. If you have a combination of *cough, fever, problems smelling, weakness, muscle aches or shortness of breath*, assume you have covid19. Don't bother getting tested. I know of many docs who don't test anymore if patient has obvious symptoms.

C2729

###### Rising Number of Cases

Our findings show that more people are getting infected with COVID-19 in many parts of the world, as shown in the following sample comments. Evidence confirms increasing numbers of COVID-19 cases in North America [88,89] and Europe [90], as well as a growing concern for vulnerable continents such as Africa [91].

There is *rapid increase in cases of COVID19 in India*...I request PM to extend the lockdown to avoid community spread.

C1325

Despite *infection cases increasing at exponential rate* doubling every 3 days, Trump pushes workers to risk their lives for economy...

C6522

###### Comparison With Other Diseases or Incidents

Our findings revealed that people compare COVID-19 with other diseases such as the flu (eg, Spanish flu and H1N1 swine flu) and SARS, and with more extreme incidents such as war. However, although some people tend to downplay the severity of COVID-19 (see C647), others think it is dangerous or frightening (see C922 and C45). Research has shown that COVID-19 has a higher transmissibility rate than SARS [92] and has killed more people than SARS and Middle East respiratory syndrome combined [93], thereby making it a highly contagious and lethal disease.

It is just *another strain of flu*. People with weak and have health problem it will affect different than people with stronger and not so much health problems. *Media is making it sound worse*...

C647

I was not at all concerned about swine flu, I was not at all concerned about Ebola, I was not at all concerned about Zika virus, but this virus *I was concerned about going all the way back to January*. If I had enough information to be concerned about this virus, then so did they. We are a month behind on dealing with this virus and there are no excuses, even a lay person like myself knew all the way back in January that this was *bigger than anything that has come before it in my lifetime*...

C922

This is a war. We need to protect ourselves and minimize unnecessary contact to avoid another Spanish flu that killed 50 million people...

C45

##### Psychosocial Issues

###### Expression of Fear and Panic Shopping

Based on our findings, people are fearful or scared about COVID-19, and although many expressed genuine fear (including those who had lost loved ones to the disease, contracted the disease, or had an infected family member), others attributed it to fear mongering that is further amplified by the media. As a result of this fear, many people engaged in panic buying to stockpile essential items so they can stay indoors and limit movements for some days or weeks to keep themselves and their families safe. The following are sample comments:

Very frightening when people who have travelled think that covid cannot affect them. Such foolish behaviour and thoughts putting all of us at risk. Those who travelled please STOP moving around and be at home.

C8887

Fear mongering through projecting number of possible deaths. The media is disgusting.

C5559

Everyone who is panic shopping is driving my family and me nuts...Everyone in our area is *panic-buying groceries*. We *can't get noodles, rice, or really any real staple foods*. We hardly have any food already. I'm kinda scared.

C2937

###### Work From Home Complaints

Furthermore, the pandemic triggered work from home (or remote work) measures to promote continuity of businesses during lockdown [94], but this may have negative implications on people’s lifestyle and well-being. For example, people found consistently working from home exhausting, boring, and distracting with kids at home. In addition, people living in countries without stable electricity and strong internet found it difficult and more costly to work from home, as they have to fuel their generators and pay more for considerably good internet connectivity. Evidence has shown that people work longer hours at home than on site due to difficulty in maintaining clear delineation between work and nonwork domains [95], thereby leading to work–family conflict and strain [96]. The following are sample comments:

Do you find *working from home exhausting?* You are not alone. Why is that and how can you combat it?

C11224

This thing of *working from home annoys*. Waking up at 4 am to have things done before the Boy wakes up. Otherwise you will spend better part of the day *watching cartoons with no work done*.

C9902

It is very clear from today's program how the government is not organized or coordinated with this issue. How should one *work from home with constant power cuts and bad internet?*

C8855

###### Frustration Due to Life Disruptions and Retrospection

Finally, people are generally frustrated about life disruptions caused by COVID-19 (which is the top issue based on our empirical findings as shown in Table 6). Based on our findings, this frustration is mostly due to decreased leisure and interaction with friends and family, authorities’ actions and inactions, and uncertainty of upcoming situations, which leads to cognitive dissonance [97], insecurity, and mental discomfort [98]. People expressed their frustrations using words reflecting anger and unhappiness or sadness, as shown in the following sample comments. Research has shown that positive emotions (eg, happiness) and life satisfaction decreased during the COVID-19 pandemic [99]. Therefore, it is unsurprising that people missed (and crave for) their prepandemic lives, in retrospection (see C377).

I am getting *more and more angry with this current situation we are in*. My favourite show has had to postpone its final episode, after already postponing series 11...

C7776

We are literally living in a time when arbitrary shit is more important than health, wellness and preservation of life. Entitlement. Ignorance. Selfishness. An untouchable mentality. Humanity at its absolute worst. *April's going to be a painful month to live through.*

C1228

I cannot wait until this whole thing is OVER. *I miss doing nails. I miss being in my element and doing the creative things I enjoy*. I have no practice hand; I have no work; I feel lost. I was just licensed in Jan!

C377

##### Social Issues

###### Wrong Societal Attitude

Our findings revealed disapproval and concerns about people’s defiance of precautionary measures or guidelines (eg, social distancing and travel guidelines) to curb the spread of COVID-19 (see C7218 and C1444) and some people’s habit of eating animals assumed to be carriers of viruses (see C4013). Research has highlighted certain factors responsible for reduced compliance with public health guidelines, such as poverty, economic dislocation, lack of compensation, and mistrust of science [100-102].

The only good thing about Coronavirus is that it will cull the *stupid people from amongst us - those that do not take it seriously and continue to gather in public*, those that *go overseas to attend weddings and other events when they know the risk*...

C7218

The public response to this crisis in the UK has been *absolutely pathetic*. Showing we are an entitled society who cannot handle being told what to do. Embarrassing that *people cannot follow simple instruction.*

C1444

Why is that someone who knows how *dangerous eating these animals* are and *still go right ahead and eat*. Is it that they do not have any sense or is it just irresponsible stupid people to do this then what are they crazy or just insane?

C4013

###### Domestic Violence

Furthermore, an increase in domestic violence incidents was reported as a result of the COVID-19–related lockdown, as shown in the following comments. Evidence already confirms the link between COVID-19 and the rising cases of domestic violence worldwide [103-107].

Cases of domestic violence in the USA has skyrocketed since the CoronaVirus forced couples to stay home together for 14 days or more.

C9900

While *domestic violence across France increased by 32%* in one week, in Paris it *rose by as much as 36%*.

C13720

What worries me more than the coronavirus, is the safety and welfare of those *stuck at home with their abusers*, the *children witnessing domestic violence* and the lonely relying on company. Staying home is not always safer.

C7910

###### Harassment

Our findings also uncovered undue harassment of people from certain cultures, races, or religious background, accusing them of spreading COVID-19. The following sample comments reveal public intimidation and racist attacks toward Chinese and Asians as well as certain Indian tribes. This aligns with evidence of widespread anti-Chinese and anti-Asian xenophobic or racist attacks, especially in the United States, both physically and on social media [108-113].

...This supermarket *refuses to sell the food to the CHINESE*! We should stop going to this supermarket! We strongly against RACISM towards Chinese people abroad! THIS MUST STOP!

C2008

Coronavirus has only taught me one thing; some *people are so racist*, especially on this platform. The amount of *hate, racist comments* and *abuse* I am seeing *Chinese/Asian people get is painful*.

C5000

Indians got racism in their blood, breath, and beyond. A *Mizo girl faced racism* in Pune as a woman kept covering a face whenever the Mizo girl passed by. A friend Naga from maternal side in Mumbai, *got called corona virus* in the middle of an empty road.

C6660

### Recommended Interventions for Addressing the Negative Issues

In this section, we suggest interventions that can help address the negative issues while drawing insights from the positive themes and relevant research evidence. This answers our RQ3.

As lockdown and physical distancing persists, people with health concerns should be able to receive medical attention without visiting a hospital. Considering the proliferation of smartphones and the current wave of global digitization, digital interventions using mobile, artificial intelligence (AI), internet of things (IoT), and virtual reality technologies have been shown to be effective for delivering remote health care (or telehealth) to patients [114-119]. This is based on our findings under the *innovative research* positive theme (see Table 7), which revealed global research efforts to create digital interventions using emerging technologies to address the health crisis caused by COVID-19. For example, mobile apps that detect mental health issues (eg, depression and anxiety) based on phone sensors (or wearable sensors) data and self-reports using machine learning and deep learning models, and then guide users through therapeutic procedures or treatments will be useful tools during and after the pandemic. In addition, these apps should allow users to book appointments with doctors, clinicians, or therapists and access remote medical advice, diagnosis, and treatments when necessary.

In addition, data-driven surveillance systems based on AI that predict the location of the next COVID-19 outbreak can enhance the effectiveness of containment efforts, thereby slowing the spread of the disease and reducing the case-fatality rate. Furthermore, the *development of curative solutions or treatments* (see Table 7) can be accelerated by leveraging machine learning and deep learning algorithms. For example, deep learning models can be used to predict chemical compounds that can halt viral replication and to suggest drugs that can be effective against the virus.

To address fitness issues during lockdown, *physical activity* (which is one of the positive themes in our results) programs or sessions with personalized feedback delivered through mobile apps would be helpful. Research has shown that smartphone-based health programs yield significant weight loss and increase physical activity [120]. There is also an urgent need to strengthen the global health care systems to cope with current and future pandemics through public and private investments in the health sector on an ongoing basis, such as provision of public health infrastructure that is robust and adequate for the target population and easily accessible and the provision of health insurance for everyone irrespective of financial status.

*Public awareness* (which emerged as the top positive theme in our findings) is also crucial for addressing negative issues arising from COVID-19 by providing timely and accurate information to people, which can be lifesaving. To reach a wider audience in an efficient manner and with less cost, public awareness can be delivered through mobile technologies, such as mobile-driven and voice-enabled conversational AI agents (or chatbots) with access to evidence-based and clinically validated resources (eg, precautionary or safety measures approved by public health agencies and organizations as well as government-approved policies or guidelines), can deliver accurate information regarding COVID-19 to people in their own native language (and in an interactive fashion) through their smartphones. These chatbots can also be made to route difficult questions to health experts for real-time feedback within the same chat session. This will help to improve people’s understanding of the disease, including how it differs from other infectious diseases, and how to protect themselves and their families from getting infected with COVID-19. In addition, people will be empowered with information required to effectively respond to fear mongering, domestic violence, and harassment. Evidence already shows the deployment of multilingual chatbots for public health awareness on COVID-19 symptoms, diagnosis, and precautionary measures [121]. Furthermore, chatbots can also respond to emergencies by contacting appropriate security agencies and emergency response teams on behalf of the users. Moreover, chatbots can deliver evidence-based therapeutic interventions to people while coordinating with specialists behind the scenes where necessary.

For people with nonsmartphone devices, public health agencies can partner with telecommunication companies to deliver COVID-19–related information directly to their phones as text messages at regular intervals. Social media is another platform through which evidence-based information can be shared with the public but may be overshadowed by fake news or false information, which is mostly shared on social media [122]. Nevertheless, official COVID-19–related channels managed by (or in conjunction with) reputable international health organizations (eg, World Health Organization) or local health authorities within the social media platforms, many of which have already been deployed, provide accurate information or updates about COVID-19 cases, fatality rates, and safety measures and guidelines [123,124]. In addition, people receive location-based updates on these channels, including emergency alerts, in a timely and effective manner.

Finally, based on our findings (see Table 7), *connection with family and friends*, *encouragement*, *spiritual support*, and *charity* can help to ease people’s frustrations, anxiety, and trauma (due to life disruptions caused by the pandemic) by addressing their emotional, physical, and spiritual needs. Evidence shows that psychological first aid and spiritual care can promote a sense of safety, calmness, self- and collective–efficacy, connectedness, and hope, as well as help people confront and overcome fear [125]. Therefore, people should endeavor to frequently communicate and follow up with loved ones (through direct voice or video calls or by using social media), encourage others in distress to stay calm and remain positive, identify people’s immediate needs and offer necessary assistance, help people find hope and meaning, and ensure the safety and comfort of vulnerable populations.

Mobile technology can play a key role in facilitating easy access to relief packages. For instance, mobile apps can be deployed with geolocation and multilingual features to help people locate the nearest food bank and charity organizations offering assistance in their geographical area. In addition, charity organizations can effectively mobilize and deliver relief items to more people, including individuals that are indisposed, based on data collected through these apps. In addition, older adults, the sick, and those in self-isolation can indicate their condition while requesting for relief so that their items can be delivered to their doorstep instead of picking it up. These apps can further integrate with other local and international charity organizations to widen the coverage of relief efforts. Recruitment of volunteers can also take place through these apps. The use data collected can be further analyzed in real time and used to predict the communities that are in dire need of assistance using machine learning or deep learning techniques.

### Limitations

In this study, we analyzed data from Twitter, Facebook, YouTube, and three discussion forums. However, people may have used other social media platforms such as Instagram and other discussion forums not covered in this study to disseminate information related to the COVID-19 pandemic. Therefore, our findings may not fully reflect the entire public’s opinion on social media with respect to the pandemic. Nevertheless, to have a reasonably broad understanding of public opinions, we analyzed over 1 million social media comments compared to only a few thousand commonly analyzed in many related studies. In addition, the thematic analysis used for theme categorization may be more robust; however, the large number of key phrases rendered this process time-consuming despite filtering out many irrelevant key phrases during experimentation. Accordingly, the saturation level and subsequent review and confirmation of the theme categories from a second reviewer and coder were introduced as an acceptable compromise.

### Conclusions

In this paper, we explored the impact of the COVID-19 pandemic on people worldwide using social media data. We analyzed over 1 million comments obtained from six social media platforms using a seven-stage NLP approach to extract candidate key phrases, which we further categorized into broad themes using thematic analysis. Our results revealed 34 negative themes, out of which 15 were *health-related issues*, *psychosocial issues*, and *social issues* related to the COVID-19 pandemic from the public perspective. The top health-related issues were *increased mortality*, *comparison with other diseases or incidents*, *nature of disease*, and *health concerns*, while the top psychosocial issues were *frustrations due to life disruptions*, *panic shopping*, and *expression of fear*. The top social issues were *harassment* and *domestic violence*. Besides the negative themes, 20 positive themes emerged from our results. Some of the positive themes were *public awareness*, *encouragement*, *gratitude*, *cleaner environment*, *online learning*, *charity*, *spiritual support*, and *innovative research*. We reflected on our findings and recommend interventions that can help address the health, psychosocial, and social issues based on the positive themes and other research evidence.

Digital interventions using emerging technologies such as mobile apps, AI, IoT, and virtual reality will play a major role in delivering remote health care (ie, telemedicine or telehealth) to people in the comfort of their homes, including empowering them to self-manage their health and wellness. This will help to curb the spread of COVID-19 and future infectious diseases since many people will stay away from hospitals (or clinics) to book appointments or see doctors (or other health care professionals) unless it is absolutely necessary to visit, thereby keeping health workers and patients safe. These technologies are also useful in providing timely and accurate information about COVID-19 symptoms, diagnosis, treatment, precautionary and safety measures and guidelines, and other relevant information to target audience worldwide. Finally, digital interventions and other interventions discussed in this paper can help address the emotional, physical, and spiritual needs of people who are traumatized or frustrated by the disruptions caused by the pandemic. They also inform governments, health professionals and agencies, and institutions on how to react to the current COVID-19 pandemic and future pandemics.

## Appendix

Multimedia Appendix 1 Top 130 negative themes and their dominance in terms of frequency of occurrence (larger size of the gray oval represents more dominance).

Multimedia Appendix 2 Sample negative themes.

Multimedia Appendix 3 Top 130 positive themes and their dominance in terms of frequency of occurrence (larger size of the gray oval represents more dominance).

Multimedia Appendix 4 Sample positive themes.

## Abbreviations

AI: artificial intelligence
API: application programming interface
IoT: internet of things
LDA: latent Dirichlet allocation
NLP: natural language processing
POS: part of speech
RQ: research question
SARS: severe acute respiratory syndrome
