# Supplementary figures and images for: Health, Psychosocial, and Social Issues Emanating From the COVID-19 Pandemic Based on Social Media Comments: Text Mining and Thematic Analysis Approach
Source: JMIR Med Inform. 2021 Apr 6;9(4):e22734. doi: 10.2196/22734 (PMC8025920; doi:10.2196/22734)

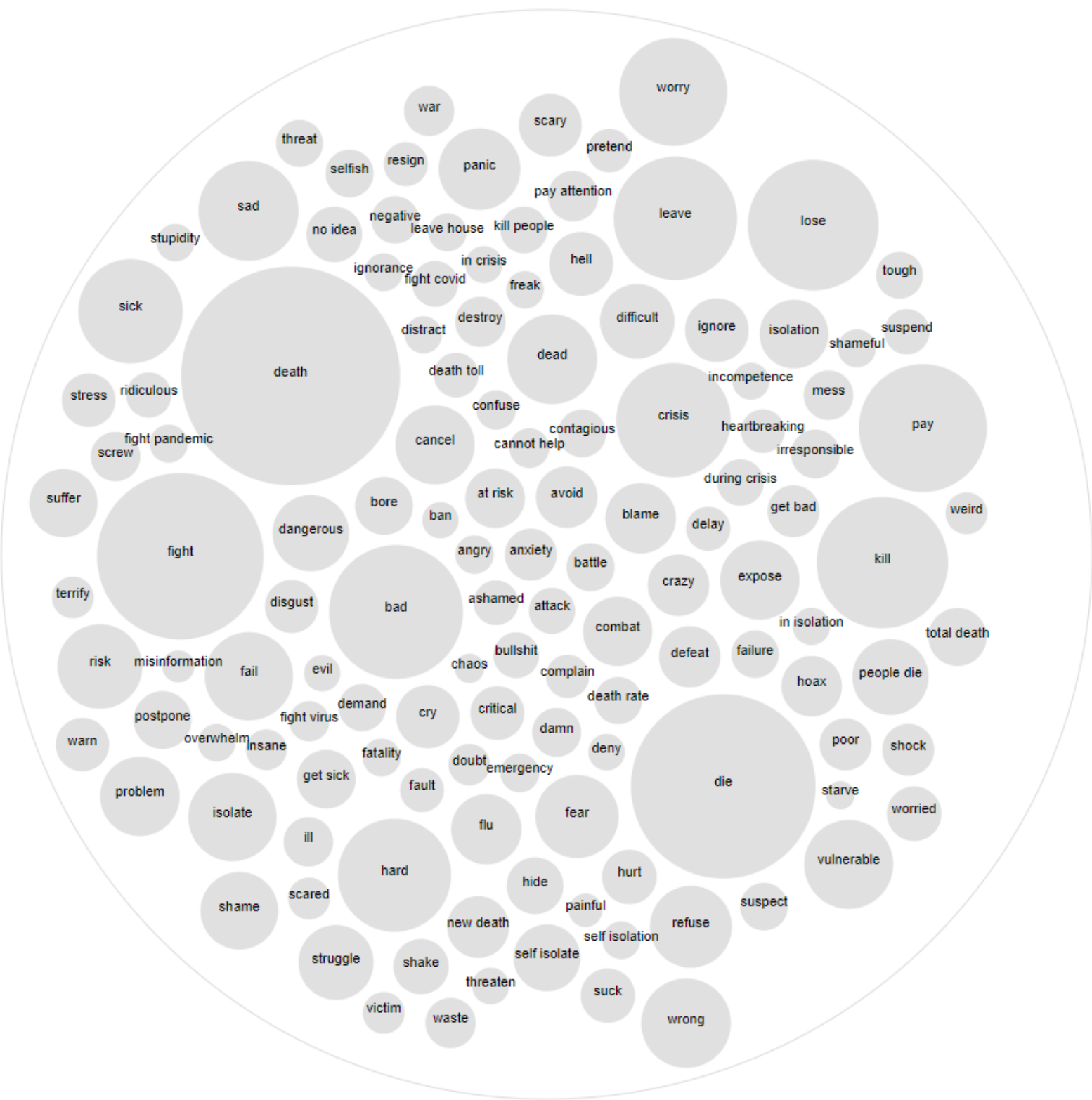

Supplement: Multimedia Appendix 1 [file medinform_v9i4e22734_app1.pdf]

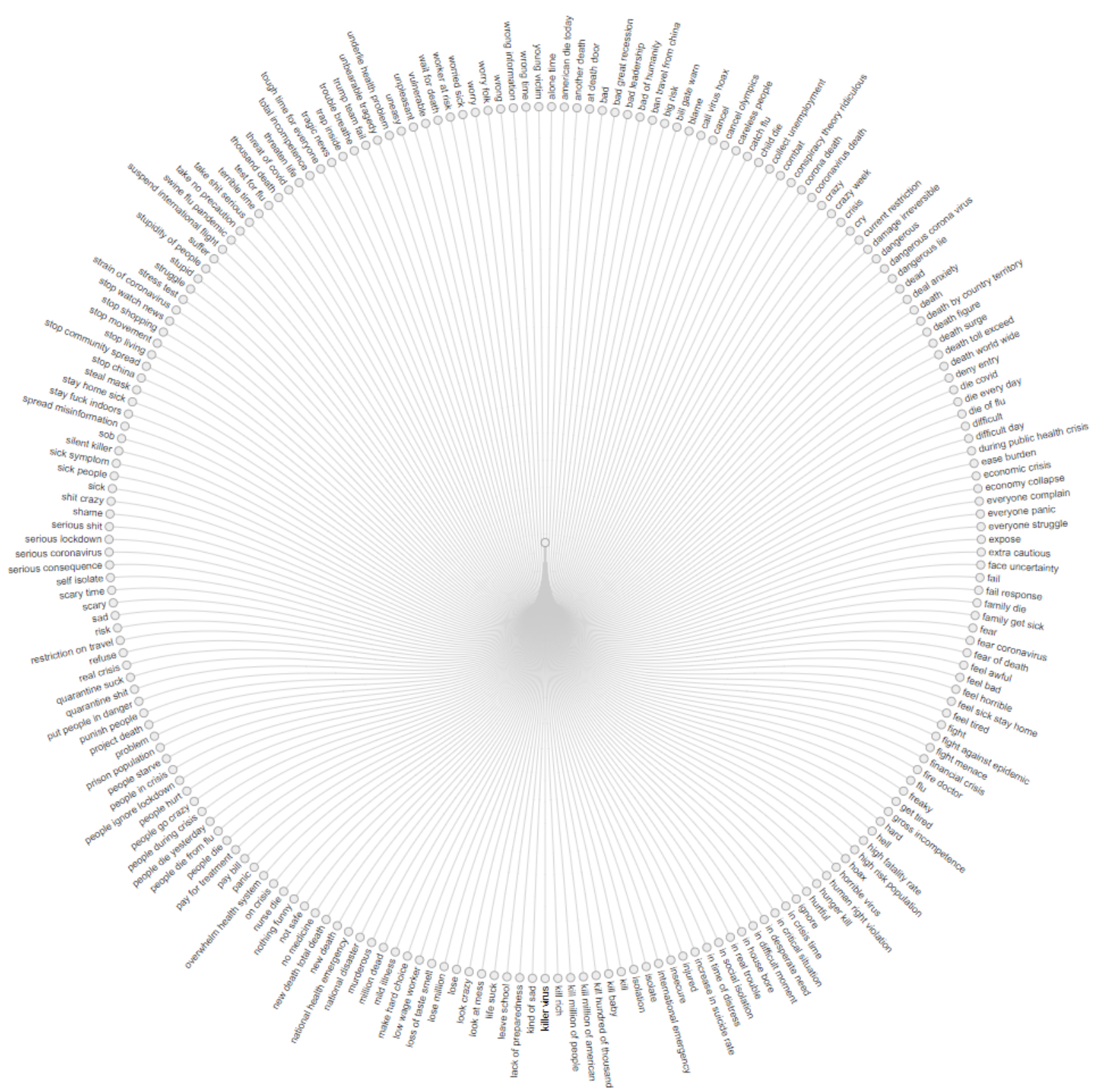

Supplement: Multimedia Appendix 2 [file medinform_v9i4e22734_app2.pdf]

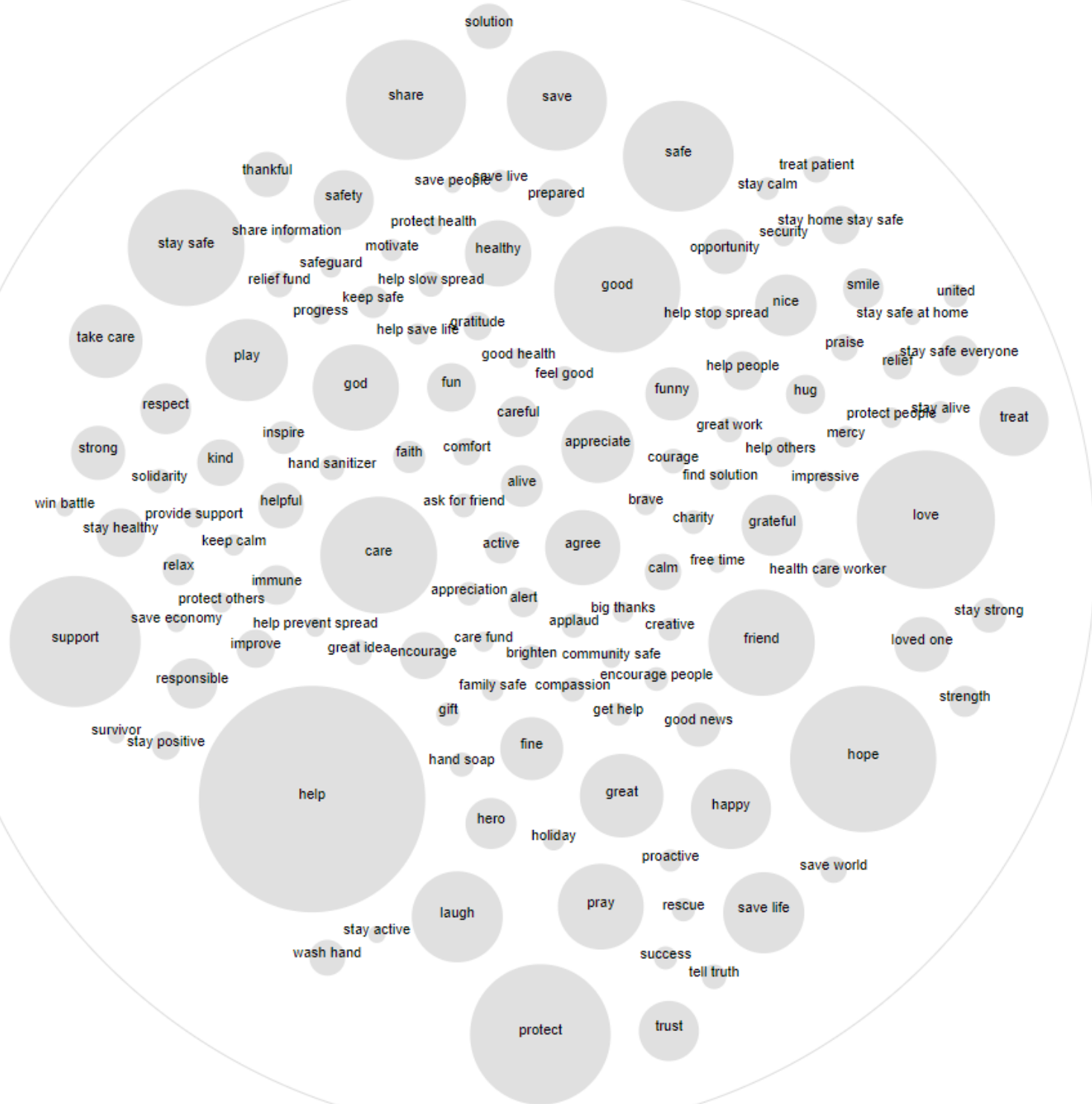

Supplement: Multimedia Appendix 3 [file medinform_v9i4e22734_app3.pdf]

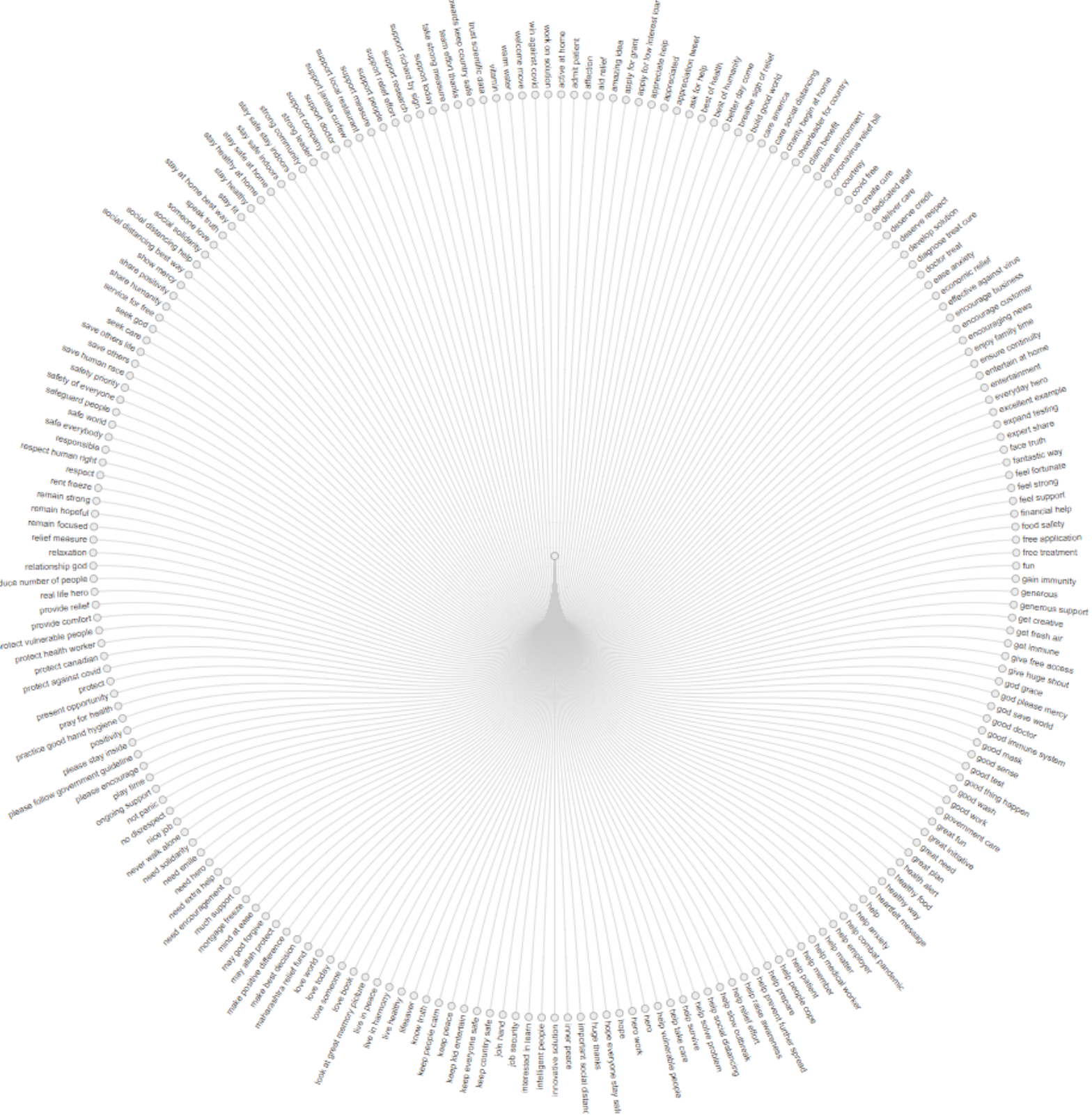

Supplement: Multimedia Appendix 4 [file medinform_v9i4e22734_app4.pdf]
